# Mindfulness Training among Individuals with Parkinson's Disease: Neurobehavioral Effects

**DOI:** 10.1155/2015/816404

**Published:** 2015-05-26

**Authors:** Barbara Pickut, Sven Vanneste, Mark A. Hirsch, Wim Van Hecke, Eric Kerckhofs, Peter Mariën, Paul M. Parizel, David Crosiers, Patrick Cras

**Affiliations:** ^1^University of Antwerp, Universiteitsplein 1, 2610 Wilrijk, Belgium; ^2^Department of Neurology, Antwerp University Hospital, Wilrijkstraat 10, 2650 Edegem, Belgium; ^3^Mercy Health Hauenstein Neurosciences, 220 Cherry Street, Grand Rapids, MI 49503, USA; ^4^Michigan State University, College of Human Medicine, Department of Translational Science and Molecular Medicine, 333 Bostwick Avenue NE, Grand Rapids, MI 49503, USA; ^5^Lab for Clinical and Integrative Neuroscience, School for Behavioral and Brain Sciences, University of Texas at Dallas, 1966 Inwood Road, Dallas, TX 75235, USA; ^6^Department of Physical Medicine and Rehabilitation, Carolinas Rehabilitation, 1100 Blythe Boulevard, Charlotte, NC 28203, USA; ^7^icoMetrix, Tervuursesteenweg 244, 3001 Leuven, Belgium; ^8^Department of Radiology, Antwerp University Hospital, Wilrijkstraat 10, 2650 Edegem, Belgium; ^9^Faculty of Physical Education and Physical Therapy, Center for Neurosciences, Vrije Universiteit Brussel, Laarbeeklaan 103, 1090 Brussels, Belgium; ^10^Department of Clinical and Experimental Neurolinguistics, Vrije Universiteit Brussel, Pleinlaan 2, 1050 Brussels, Belgium; ^11^Department of Neurology and Memory Clinic, Ziekenhuis Netwerk Antwerpen, Lindendreef 1, 2020 Antwerp, Belgium; ^12^Born Bunge Institute, Universiteitsplein 1, 2610 Wilrijk, Belgium

## Abstract

*Objective*. To investigate possible neurobehavioral changes secondary to a mindfulness based intervention (MBI) training for individuals living with Parkinson's disease (PD). *Background*. In the context of complementary medicine, MBIs are increasingly being used for stress reduction and in patient populations coping with chronic illness. The use of alternative and complementary medicine may be higher in patients with chronic conditions such as PD. However, behavioral effects of mindfulness training in PD have not yet been reported in the literature and this points to an unmet need and warrants further examination. *Methods*. A total of 27 out of 30 PD patients completed a randomized controlled longitudinal trial. Questionnaires and the UPDRS I–IV were obtained at baseline and 8-week follow-up. *Results*. Significant changes after the MBI were found including a 5.5 point decrease on the UPDRS motor score, an increase of 0.79 points on Parkinson's disease questionnaire (PDQ-39) pain item, and a 3.15 point increase in the Five Facet Mindfulness Questionnaire observe facet. *Conclusions*. To the best of our knowledge, this is the first quantitative analysis of neurobehavioral effects of MBI in PD.

## 1. Introduction

Parkinson's disease (PD) is a chronic disease consisting of motor and nonmotor symptoms. Dopaminergic substitution remains the golden standard of treatment. Despite current advances in the understanding of the complexities of the disease process itself and the mechanisms of pharmacological therapy, no established secondary neuroprotective or curative interventions are available to offer to people living with PD. People living with PD may be faced with and often describe a worsening of motor symptoms, such as tremor and freezing of gait in stressful situations. They also experience nonmotor symptoms such as depression and anxiety, which stressful situations may exacerbate. It has been suggested that nonmotor features of PD, including cognitive impairment, depression, and anxiety, impact health related quality of life more than other determinants including physical parameters [1]. It has been hypothesized that stress contributes to the development of PD and that depression may exacerbate the symptoms of PD (for a review, see Hemmerle et al. [2]).

As such, absence of a perspective to improve may motivate patients to utilize complementary and alternative medicine (CAM) approaches such as mindfulness training. The use of CAM is reportedly higher in patients with chronic conditions in general [3] and neurologic conditions in particular [4]. Worldwide it has been estimated that between 25% and 76% of people with PD utilize CAM [5]. In the USA, a 2001 survey of ambulatory clinics reported that almost 40% of the patients with PD had used CAM [6]. Mindfulness based stress reduction (MBSR) was first conceptualized and used in The Stress Reduction Clinic by Kabat-Zinn as a complementary intervention in a hospital setting to serve as a referral service to physicians and other health care providers for patients with chronic pain [7, 8] or patients who were not responding sufficiently to treatment or who were dissatisfied with conventional therapies [9].

Patients may ask advice of their allopathic health care practitioners regarding the use of mindfulness training as a CAM approach. However, health care providers may be limited in offering guidance to interested patients due to scarce efficacy data. To help address this, the aim of our study was to investigate possible behavioral changes secondary to a mindfulness based intervention (MBI) for individuals living with Parkinson's disease.

## 2. Materials and Methods

### 2.1. Participants

For participant inclusion to the study, experienced neurologists verified all the patients for the following set of criteria: (1) diagnosis of PD according to the UK Brain Bank Criteria, (2) patients in Hoehn and Yahr stages I–III, (3) lack of features suggestive of atypical parkinsonism, (4) exclusion of usage of neuroleptics or other drugs that induce parkinsonism 60 days prior to inclusion, (5) optimally treated with PD medication and unlikely to require anti-PD medication adjustments in the next 3 months, (6) stable dose of all medications for 30 days prior to inclusion, (7) lack of cognitive dysfunction as evidenced by the Montreal Cognitive Assessment Test (MoCA) (score ≥ 26), (8) no known unstable or life threatening concomitant disease, (9) no previous mindfulness training, and (10) commitment to attend all eight MBI classes and perform the prescribed daily homework.

A total of 30 individuals with PD were randomized 1 : 1 into mindfulness based intervention (MBI) or usual care (UC) arms of 15 participants each. Randomization was conducted by a blinded investigator. The groups were comparable at baseline with respect to age, gender, and disease severity (Table 1). Of these, 27 completed the controlled longitudinal trial. Fourteen participated in an 8-week MBI and 13 received UC alone consisting of regularly scheduled visits to a movement disorders specialist or neurologist or as needed. Two patients, allocated to the UC group, dropped out for personal reasons (not considered relevant to the study) during follow-up and one patient of the MBI group discontinued study participation due to acute back pain. The medication remained stable in 26 out of 27 patients. During the study, one patient in the UC group had an increase of 50 mg levodopa and 50 mg sertraline, as deemed necessary by the subject's general practitioner.

### 2.2. Mindfulness Based Intervention

The intervention consisted of a mindfulness based intervention (MBI) closely following mindfulness based stress reduction (MBSR) as described by Kabat-Zinn [10]. The MBI consisted of 2.5 h meetings on eight consecutive weeks without the one-time full-day session in the sixth week of practice as described in MBSR. In order to facilitate the development of the capacity for mindfulness, the key components of formal mindfulness exercises in meditation, yoga, and the body scan were practiced during the eight sessions. To facilitate the integration of mindfulness into daily life, instructions were given on how to practice and integrate mindfulness in everyday activities such as eating, walking, or performing household chores. Participants also received instructions in using mindfulness for coping with stress in daily life. Audio recordings containing 45 min guided mindfulness exercises (meditation, yoga, and the body scan) were given with instructions for daily home practice corresponding to the course sequence. Time spent in mindfulness practices, as reported by the participants, was recorded weekly for each participant. The MBI was conducted by two experienced mindfulness teachers. All participants in both the UC and the MBI groups received regular, optimal medical care during the duration of the study. UC patients were offered the MBI two months after the study completion.

### 2.3. Questionnaires

Patient questionnaires and the Unified Parkinson's Disease Rating Scale (UPDRS) [11] were conducted at baseline and after the 8-week training. The investigator scoring, including UPDRS motor scoring, was performed by a movement disorders specialist who was blinded as to study allocation arm of the participant while the participants were experiencing on-effects of their medication.

The UPDRS is divided into four subscales: subscale I (nonmotor experiences of daily living), subscale II (motor experiences of daily living), and subscale IV (motor complications) which are patient and caregiver-oriented questionnaires. UPDRS Subscale III (motor examination) is an objective assessment of the patient's motor abilities. Each question has a 5-point scale, where 0 means an absence of symptoms and 4 represents severe symptoms.

In the* Five Facet Mindfulness Questionnaire* (FFMQ) of 39 items, five domains of mindfulness “skills” are assessed: observing, describing, acting with awareness, nonjudging of inner experience, and nonreactivity to inner experience [12]. Adding up the domains results in a total score of an approximation of mindfulness “skills.” Items are rated on a 5-point Likert-type scale from “never or very rarely true” to “very often or always true.” The subscales of the Dutch FFMQ have been shown to have good internal consistencies.

The* PDQ-39* is a subjective questionnaire to assess QoL in patients with PD [13]. The questionnaire consists of thirty-nine questions, which are divided into eight subscales (mobility, ADL, emotional well-being, stigma, social support, cognition, communication, and bodily discomfort). Each question has a range from 0 (no problem) to 100 (continuous problem/unable to do it).

The* Beck Depression Inventory* (BDI) is a questionnaire to evaluate the severity of depressive mood states. It scores components like hopelessness and guilt feelings, as well as fatigue and other physical symptoms. It consists of 21 questions, rated between 0 (no symptom impact) and 3 (maximum symptom impact) with a maximum score of 63 [14].

All participant-reported outcome measures were entered into a database by personnel blinded to group assignment. All investigator rated scales were administered by a blinded assessor. Standard protocol approvals, registration, and patient consent forms were approved by the Institutional Review Board of the Antwerp University Hospital, Antwerp, Belgium. The study was registered at ClinicalTrials.gov (#NCT01607697). All participants gave written informed consent.

### 2.4. Statistical Analysis

A repeated measures ANOVA with as within subjects variable the questionnaires and between subjects variable the group (Mindfulness Based Intervention Group vs Usual Care Group) was carried out on data obtained from the questionnaires completed at baseline and after 8-week follow-up. Attendance to the training sessions and amount of home practice time were recorded.

## 3. Results

**Figure 1:**
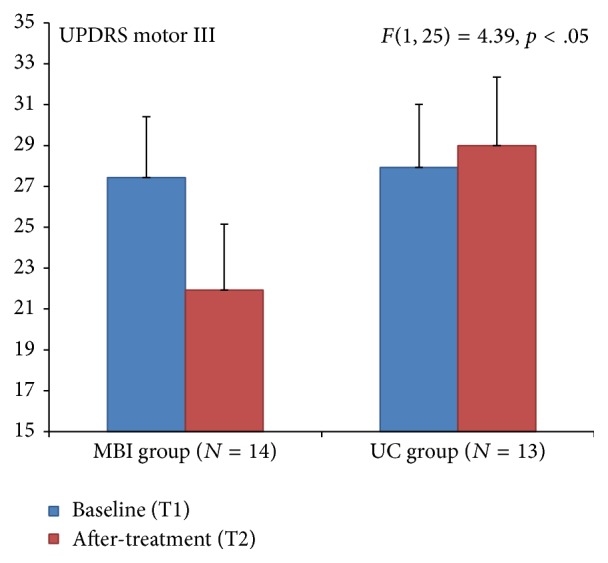
Unified Parkinson's Disease Rating Scale (UPDRS) for motor change showed a significant effect for the MBI group.

Significant change was obtained for the UPDRS motor III score (*F *= 4.39, *p* < .05), demonstrating motor changes for the MBI group (pre-M = 27.43 versus post-M = 21.93), while no significant changes could be obtained for the UC group (pre-M = 27.92 versus post-M = 29). This revealed that after the MBI a decrease of 5.5 (20.05%) points was found on the UPDRS motor score (see Figure 1). No significant effects were obtained for the other subscales on the UPDRS.

**Figure 2:**
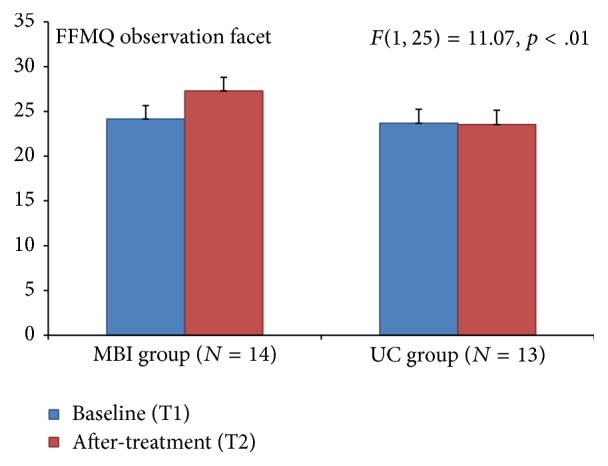
Five Facet Mindfulness Questionnaire (FFMQ) for observation facet showed a significant effect for the MBI group.

A significant interaction effect (*F *= 11.07, *p* < .01; see Figure 2) was obtained for the FFMQ observe facet of the scale indicating that for the MBI group there was a significant increase after treatment (M = 27.29) in comparison with before treatment (M = 24.14). For the UC group, no difference was obtained between before (M = 23.69) and after treatment (M = 23.54). No significant effect was obtained for the other facets of the FFMQ. This indicates a 3.15- (13.04%-) point increase in the Five Facet Mindfulness Questionnaire observe facet.

**Figure 3:**
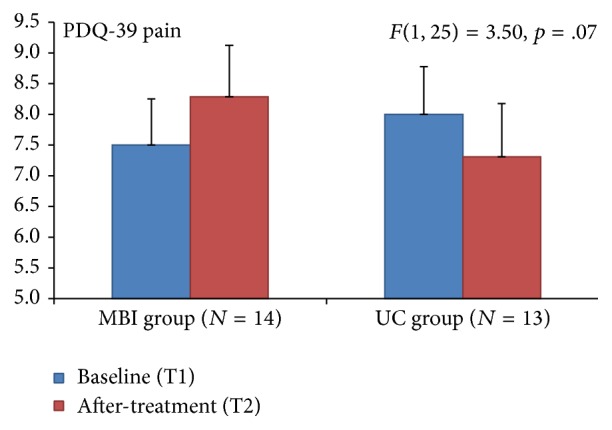
Parkinson's Disease Quality of Life (PDQ-39) showed a marginal significant effect for the MBI group.

A marginal significant interaction effect (*F *= 3.50, *p* = .07; see Figure 3) was obtained for Parkinson's disease questionnaire (PDQ-39) pain score, suggesting an increase of 0.79 (10.53%) points for the MBI group and decrease of 0.69 (8.63%) for the UC group.

The total PDQ-39 score and the Beck Depression Inventory did not reach significance.

Attendance rate at the training sessions of the MBI group was 97.3%. Participants reported spending a total of 32,244 min performing the key components over the 8-week training with an average of 55 min per day. The average time spent on practicing each key component was 47.45% on meditation, 30.76% on yoga, and 21.79% on body scan.

## 4. Discussion

Differences in three behavioral measures between the MBI and the UC groups were observed in this study population of people with Parkinson's disease after following 8 weeks of a mindfulness based intervention training. Significant changes after the MBI were found including a 20.05% decrease on the UPDRS motor score, an increase of 10.53% on Parkinson's disease questionnaire (PDQ-39) pain item, and a 13.04% increase in the Five Facet Mindfulness Questionnaire observe facet.

The single-blind movement disorders specialist rated UPDRS motor score improved 5.5 points in the MBI group and the UC group had a 1.08-point worsening. A change of 5 points has been suggested to be the most appropriate cut-off score for a clinically meaningful improvement after an intervention [15] and within the moderate range of clinically important differences in UPDRS motor score [16]. In our study, a single-blind wait list control paradigm was used. The placebo effect should be considered in the interpretation of the significant changes in motor function as measured on the UPDRS in this study. Placebo effect in PD is a well-known phenomenon and placebo induced release of endogenous dopamine in the striatum has been reported [17]. The strength of belief of improvement can directly modulate dopamine release in patients with PD [18]. Positive expectations regarding mindfulness abound in the popular press and this has important implications for the interpretation of this study and for the design of future clinical trials using mindfulness as an intervention in PD. At the time of the study design, no generally accepted validated control group paradigm had been published in the literature.

The FFMQ is a widely used self-report rating scale in mindfulness based intervention research which aims to measure mindfulness skills through five subscales: nonreactivity to inner experience, observing, acting with awareness, describing, and nonjudging of experience [12]. The observing facet of the FFMQ refers to noticing or attending to internal and external experiences. Mean score of the MBI group increased by 3.15 points, and the score in the UC group decreased by 0.15 points after the intervention. It has been hypothesized that the mindfulness “skill” is related to high levels of observing. Meditators who have a high total score on the FFMQ consistently have high observing-facet scores. The converse is found in nonmeditators [19].

Pain reported on the PDQ-39 increased modestly but significantly in the MBI group by 0.79 points and decreased in the UC group by 0.69 points. Minimally clinically important differences in PDQ-39 of 2 points may be defined as the difference that is subjectively meaningful to the patient [20] which neither study arm achieved. However, when taken together with the increase in FFMQ observe findings in the MBI group, the change in the reporting of pain in this study population may be of interest. In mindfulness training, participants are asked to actively observe sensations in the body. This could be the underlying cause of an increased reporting of pain in our population although this stands in contrast to reports that mindfulness has been associated with an attenuation of pain reporting in other populations [21]. Pain is a troublesome, frequently reported and complex, multifactorial nonmotor symptom in PD and the exact relationship between the disease and pain remains to be elucidated [22]. Perhaps future examination of three key components of mindfulness training and their impact on PD may lead to “dosing” (determining the appropriate use of) each key component to lead to better outcomes. These studies may find a basis in recent neuroimaging findings that have begun to provide insights into the neurobiological mechanisms associated with the practice of mindfulness. Mindfulness training has been shown to promote increases in regional brain gray matter density (GMD) on brain magnetic resonance imaging (MRI) in heterogeneous populations seeking stress relief [23]. Pickut et al. recently showed significant changes in GMD in a group of people with PD after 8 weeks of mindfulness training in brain areas possibly relevant to the pathophysiology of PD [24].

The degree of participation of the individuals in this study was high demonstrating the adherence to a MBI in people with PD. The attendance rate at the training sessions of the MBI group was 97.3% and the level of self-reported engagement with home practice averaged 55 minutes per day. As a group, participants reported spending time practicing each key component as 47.45% on meditation, 30.76% on yoga, and 21.79% on body scan.

## 5. Conclusions

To the best of our knowledge, this is the first explorative quantitative analysis of behavioral effects of mindfulness training in people living with Parkinson's disease. We conducted a longitudinal single-blind randomized controlled trial on a diverse population of individuals with PD who were not preselected based on an a priori hypothesis of who might benefit most from mindfulness training. PD induces an increasing loss of both motor and nonmotor functions over time in the affected individual. Currently, there is no cure or established secondary neuroprotection for PD. Even with advances in symptomatic treatment, there is a need for adjuvant person-centered therapeutic approaches to help improve the well-being of people who are faced by this disease in daily life. The role of nonpharmacological interventions in the treatment of motor and nonmotor symptoms of PD is being established. The integration of complementary and alternative medicine (CAM) approaches, including MBIs, in medicine may offer people with PD the possibility to influence personal health through a greater locus of control regarding their disorder and thus restoring some degree of self-determination and empowerment during the disease process [25].

Mindfulness training, as taught by qualified and experienced teachers, may offer a more participatory medicine, empowering the individual by engagement to learn how to strengthen internal resources to help cope with chronic disease. Mindfulness training may help to restore some degree of self-determination in the experience of living with PD. This is in line with person-centered research that employs scientific methods that are holistic, integrated, and transdisciplinary [26].

Caution must be used in the interpretation of the results presented here and we acknowledge some limitations of this study. First, the existence and longevity of behavioral changes need to be established. Thus, future larger, randomized controlled longitudinal studies are warranted to verify our results. Secondly, this study lacks an active validated control group to identify mindfulness training specific effects from general effects of a group intervention such as social support or positive expectancies that may play a role in the placebo effect.

The findings of this study may demonstrate the feasibility of and adherence to a program of mindfulness training for individuals living with Parkinson's disease.

## Figures and Tables

**Table 1:** Baseline demographic characteristics by intervention status.

Characteristic	MBI (*n* = 14)	UC (*n* = 13)	Overall (*N* = 27)	Comparison between groups
Male/women	7 (50%)/7 (50%)	7 (54%)/6 (46%)	14 (52%)/13 (48%)	*χ* ^2^ = .04, n.s.
Age	M = 61.4 years, Sd = 11.3	M = 62.2 years, Sd = 6.6	M = 61.8 years, Sd = 9.1	*t* = −.20, n.s.
H&Y	M = 2.1, Sd = .3	M = 2.2, Sd = .5	M = 2.2, Sd = .4	*U* = 74, n.s.

MBI: mindfulness based intervention group; UC: usual care group; H&Y: Hoehn and Yahr.
